# Long-term outcome of catheter ablation of left fascicular ventricular arrhythmias

**DOI:** 10.1007/s10840-025-02116-6

**Published:** 2025-08-14

**Authors:** Ilaria My, Fabian Moser, Fabian W. Loeck, Julius Obergassel, Laura Rottner, Marc D. Lemoine, Paulus Kirchhof, Daniel Steven, Arian Sultan, Stephan Willems, Christian Meyer, Bruno Reissmann, Andreas Rillig, Andreas Metzner, Feifan Ouyang

**Affiliations:** 1 Department of Cardiology, University Heart & Vascular Center Hamburg, University Medical Center Hamburg-Eppendorf, Martinistraße 52, 20246 Hamburg, Germany; 2 Department of Electrophysiology, University Heart Center Cologne, Cologne, Germany; 3 Asklepios Clinic St. Georg, Hamburg, Germany; 4 Division of Cardiology/Angiology/Intensive Care, cNEP, Cardiac Neuro- and Electrophysiology Research Group, EVK Duesseldorf, Duesseldorf, Germany

**Keywords:** Idiopathic arrhythmias, Fascicular, Ventricular tachycardia, Conduction system

## Abstract

**Background:**

Due to their low prevalence in Europe, data on optimal treatment of ventricular arrhythmias (VAs) involving the left ventricular conduction system are scarce.

**Aim:**

To report on clinical and procedural characteristics and long-term outcomes of European patients undergoing catheter ablation of primary ventricular complexes (PVCs) and ventricular tachycardias (VTs) involving the left ventricular conduction system.

**Methods and results:**

This study includes 27 retrospectively identified Caucasian patients (10/27 (37%) women, median age 44.5 (IQR 33–55.75) who underwent electrophysiological examinations at a tertiary ablation center over a period of 14 years (between 2009 and 2022). Mapping and ablation were performed via transaortic and/or transseptal approach. Post-ablation follow-up (FU) was performed via regular Holter-ECGs and clinical evaluations, or via structured FU within the prospective TRUST registry (ClinicalTrials.gov Identifier: NCT05521451). VAs were located in the left posterior fascicle (LPF) in 21/27 patients (78%), the left anterior fascicle (LAF) in 4 (15%), and the upper septum (US) in 2 (7%). Among patients presenting with arrhythmias involving the LPF, the majority (12/21, 57%) presented with sustained VTs, and 9/21 (43%) experienced PVCs/non-sustained (ns)-VTs. In contrast, among those with arrhythmias involving the LAF, the predominant clinical presentation was PVCs or ns-VTs (3/4, 75%). Of the two patients with arrhythmias involving the US region, one (50%) presented with PVCs and ns-VTs, and the other (50%) with sustained VT. Ablation was acutely successful in 24 patients (89%) with a procedure time of 130 ± 49 min. Three of the 27 patients (11%) underwent re-ablation due to early arrhythmia recurrence. No procedure-related complications occurred except left fascicular posterior block in four (15%) and a complete left bundle block in one patient (4%). Arrhythmia-free survival after a median follow-up of 30 (IQR 14–62) months was 73%.

**Conclusion:**

VAs predominantly presented as tachycardia involving the posterior fascicle and as PVCs involving the anterior fascicle and both can be treated by catheter ablation with favorable long-term clinical outcome.

**Graphical abstract:**

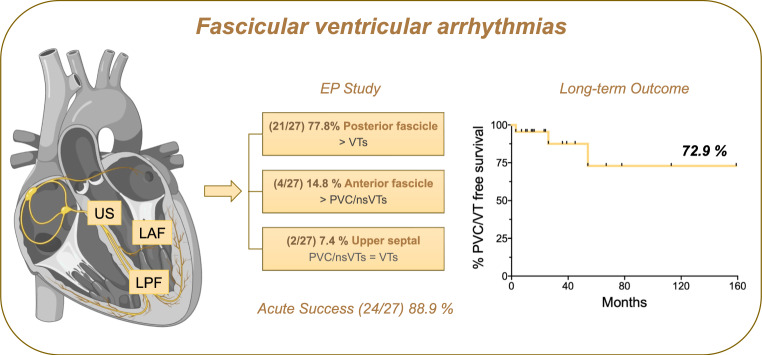

## Introduction

Fascicular ventricular tachycardia (FVT) was first identified by Zipes et al. in 1979 [1]. Two years later, Belhassen et al. demonstrated that verapamil could effectively terminate FVT episodes, providing a key diagnostic and therapeutic option for managing this condition [2].

FVT account for less than 10% of all ventricular tachycardias (VTs) and is recognized as the most common form of idiopathic ventricular tachycardia arising from the left ventricle (LV) [3]. This arrhythmia shows a marked male predominance, often presenting in young adults aged 15 to 40 who have structurally normal hearts [4, 5]. Moreover, FVT is more commonly observed in Asian populations compared to Western countries, suggesting potential genetic or demographic influences [4]. Patients with FVT often present with recurrent episodes of palpitations, dizziness, or syncope. The arrhythmia can be sustained or non-sustained and is often triggered by physical exertion or emotional stress [6].

FVT is classified as a macro re-entry tachycardia, characterized by the involvement of normal Purkinje system and the abnormal Purkinje tissue with decremental conduction properties and verapamil-sensitivity [5]. Despite numerous studies, the complete structure and dynamics of the re-entry circuit in FVT remain only partially understood and are not fully delineated [7, 8]. Myocardial tissue bridges between antegrade and retrograde Purkinje fibers have been also hypothesized [8, 9].

Three distinct subtypes of FVT have been classified based on their electrocardiographic characteristics [7]:

- Left posterior FVT (90–95% of cases)
- Left anterior FVT (5–10% of cases)
- Upper septal FVT (less than 1% of cases)

Besides VTs, primary ventricular complexes (PVC) can also occur from the left ventricular conduction system. Current clinical practice guidelines [10, 11] give a class I recommendation for catheter ablation of VA originating from the left posterior fascicle. However, most of the supporting evidence in the literature is derived from studies in Asian populations [4].

### Study aim

This study aims to provide a comprehensive analysis of clinical, mapping, and ablation characteristics, as well as long-term outcomes of patients after ablation of VA involving the left conduction system in a European population.

## Methods

### Patient selection

From May 2009 to December 2022, patients who underwent catheter ablation for PVC/VTs at the University Heart and Vascular Center in Hamburg (Germany) were retrospectively screened to identify a cohort of patients presenting with arrhythmias originating in proximity to the left ventricular conduction system.

Patient demographics, PVC burden, presence of sustained or non-sustained (ns)-VT, echocardiographic parameters, clinical history and procedural details, EP diagnosis, complications, and follow-up information were systematically collected through the TRUST registry. The study was performed in accordance with the Declaration of Helsinki of 2013, and it was approved by the ethics committee in Hamburg (NCT05521451, 2020–10066-BO).

### Mapping and ablation procedure

Antiarrhythmic drugs (AADs) were discontinued for at least five half-lives prior to the EP study. The procedures were performed by experienced operators in deep sedation applying propofol, fentanyl, and facultative midazolam. Three femoral venous accesses were obtained, and diagnostic catheters were positioned in the coronary sinus and right ventricle. Mapping was performed via a transaortic via the right femoral artery or/and transseptal approach at discretion of the operator. Once accessed the left chambers, intravenous heparin was administered to maintain an activated clotting time over 300 s.

An irrigated catheter (Thermocool or SmartTouch, Biosense Webster; TactiCath or TactiFlex, Abbott) was used for mapping and ablation. Intracardiac echocardiography was not employed in any of the procedures. Bipolar and unipolar electrograms were filtered at 30–500 Hz and 0.05–500 Hz, respectively. Intravenous isoproterenol at a rate of 1–5 μg/min and programmed pacing with up to three extra stimuli were used to provoke the PVC/VTs if necessary. Activation mapping of the PVC/VTs was performed using a 3D mapping system (CARTO 3, Biosense Webster or Ensite NavX, Abbott, Inc.).

Under sustained VT, the earliest Purkinje (P2) potential was initially targeted if a diastolic potential with sharp, high-frequency potential (P1) was not identified. In the absence of sustained PVC/VT, careful mapping during sinus rhythm was performed, identifying abnormal retrograde Purkinje potentials within the Purkinje fiber network. As described by Ouyang et al. [5], the earliest retrograde Purkinje potentials (retro-PPs) are critical for the ILVT substrate which can serve as effective targets for successful ablation [5, 11, 12]. The site of earliest Purkinje potential was used for the targeted ablation in patients presenting only with PVCs.

### Ablation settings

Power-controlled irrigated radiofrequency energy was delivered at the site of the earliest Purkinje potential during VES/VT, or at early retrograde Purkinje potentials during sinus rhythm. Power output was titrated from 20 up to 45 W with flow rate of 17–30 ml/min and maximal temperature of 43 °C.

### PVC/VT categorization

PVCs/VTs were categorized based on electrocardiographic QRS morphologies and to 3D mapping-guided anatomic localization along the LV conduction system. Origins were the anatomical locations where successful ablations were achieved.

- Left posterior fascicular (LPF) PVC/VTs, whose QRS morphology exhibits a right bundle branch (RBBB) configuration, a left axis deviation, and a localization along the posterior fascicle
- Left anterior fascicular PVC/VTs, whose QRS morphology exhibits an RBBB configuration, right-axis deviation, and a localization along the anterior fascicle
- Upper septal fascicular PVC/VTs, whose QRS morphology exhibits a narrow QRS configuration, normal or right-axis deviation, and a localization at the upper septal area.

### Clinical outcome assessment

Endpoint of RF ablation was sustained suppression of PVCs/VTs and/or non-inducibility of tachyarrhythmia for at least 30 min after the last application, with isoprenaline infusion and programmed stimulation. In case of non-inducibility and presence of retro-PP during SR, the potentials were eliminated as the procedural endpoint.

Major complications were defined as cerebral/systemic embolism, pericardial tamponade, third-degree atrioventricular block, bleeding requiring transfusion and surgical repair, infection, myocardial infarction, and death. Hematomas at access sites and pericardial effusions > 5 mm were considered minor complications.

Patients were evaluated at clinical visits 3 to 6 months after the initial procedure. Those experiencing symptoms suggestive of recurrence were advised to undergo 24-h Holter monitoring. Long-term follow-up was conducted via regular Holter-ECGs and clinical evaluations, or via structured follow-up within the prospective TRUST registry.

Long-term success was defined as a significant reduction in PVC burden and the absence of VTs as assessed via Holter-ECGs.

### Statistical analysis

All categorical variables, such as patient and procedural characteristics, are reported as absolute and relative frequencies. Continuous variables were tested for normal distribution using the Shapiro–Wilk test. They were reported as mean ± standard deviation in case of normal distribution and as median and interquartile range (IQR) (first quartile, third quartile) otherwise. Curves of long-term success were created using the Kaplan–Meier method. Data were summarized in an Excel sheet and statistical analyses were performed using GraphPad Prism versions 8 and 10.

## Results

### Baseline patient characteristics

A total of 27 patients undergoing EP studies for PVCs/ns-VTs or sustained VTs involving the fascicular conduction system were included in the study. All patients were Caucasian European. Ten of the 27 (37%) patients were women, median age was 44.5 (IQR 33–56). Twenty-two of the 27 (81%) patients presented with a preserved LVEF and 5/27 (18%) had a LVEF < 50% at baseline. Among the 27 patients, 13 (48%) presented with PVCs or ns-VTs, of whom 9 (69%) were female. The remaining 14 patients (52%) exhibited sustained VTs, with a predominant male representation (13/14, 93%) (Table 1).

**Table 1:** Baseline patient characteristics

Patients	N = 27
Caucasian ethnicity	27 (100)
Female gender, n (%)	10 (37)
Age (years), median (Q1–Q3)	44.5 (33–55.75)
Body mass index (kg/m^2^), median (Q1–Q3)	25.6 (23.2–29.3)
Arterial hypertension, n (%)	14 (51.8)
Coronary artery disease, n (%)	3 (11.1)
Preserved LVEF, n (%)	22 (81.5)
LVEF < 50%, n (%)	5 (18.5)
Structural heart disease, n (%)	6 (22.2)
Dilated cardiomyopathy	1 (3.7)
Ischemic cardiomyopathy	1 (3.7)
Valvular cardiomyopathy	1 (3.7)
Myocarditis	2 (7.4)
Other	1 (3.7)
Antiarrhythmic medication prior ablation, n (%)	23 (85)
Amiodarone	0 (0)
Betablocker	11 (40.7)
Flecainide	2 (7.4)
Verapamil	9 (33.3)
Presentation with PVCs/ns-VTs, n (%)	13 (48.1%)
Female gender	9/13 (69.2%)
Presentation with sustained VTs, n (%)	14 (51.9%)
Male gender	13/14 (92.9%)

*LVEF* left ventricular ejection fraction, *PVCs* premature ventricular complexes, *ns-VTs* non-sustained ventricular tachycardia, *VTs *ventricular tachycardia, *Q1* first quartile, *Q3* third quartile

### Procedural characteristics

**Fig. 1:**
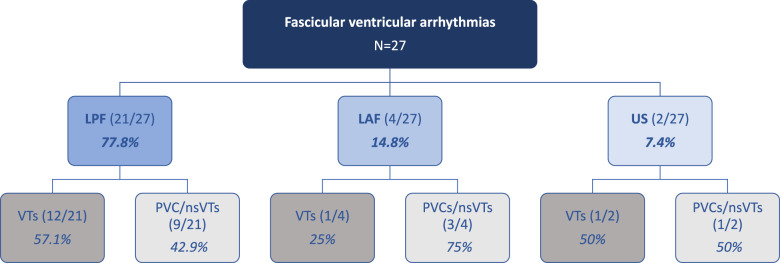
Flow chart of fascicular ventricular arrhythmias included in the study, with a focus on the anatomical distribution and the arrhythmias entity in the different localizations. LPF, left posterior fascicle; LAF, left anterior fascicle; US, upper septal; VTs, ventricular tachycardias; PVC/ns-VTs, premature ventricular contractions/non sustained ventricular tachycardias

In 21/27 patients (78%), the arrhythmia involved the left posterior fascicle (LPF). The left anterior fascicle (LAF) was implicated in four patients (15%), while the upper septal (US) region was identified as the origin in two patients (7%). Among patients presenting with arrhythmias involving the LPF (*n *= 21), the majority (12/21, 57%) presented with sustained VTs, and 9/21 (43%) experienced PVCs/ns-VTs. In contrast, among those with arrhythmias involving the LAF, the predominant clinical presentation was PVCs or ns-VTs (3/4, 75%). Of the two patients with arrhythmias involving US region, one (50%) presented with PVCs and ns-VTs, and the other (50%) with sustained VT (Fig. 1).

**Fig. 2:**
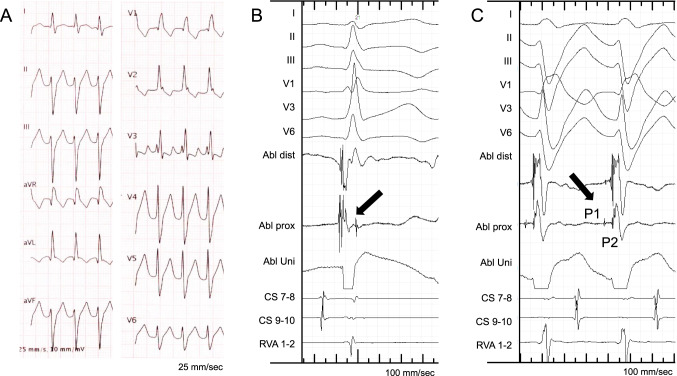
Representative case of fascicular ventricular tachycardia. A 22-year-old man presenting with a fascicular ventricular tachycardia. **A** 12-lead ECG of the tachycardia presenting a right bundle branch block and left axis deviation. **B** Intracardiac retrograde Purkinje potentials during sinus rhythm (arrow). **C** Intracardiac Purkinje signals during tachycardia (P1 and P2, arrow)

At the start of the EP study, only two patients (7%) presented with sustained VT. VT induction was successful in 16 patients (59%) applying isoprenaline and/or programmed ventricular stimulation. Detailed 3D mapping of the LV was performed in all patients. No multiple-electrode catheter located along the septum was used. When PVC/VTs were present under sedation, activation mapping was conducted to identify the earliest Purkinje signals. In case of absence of inducible arrhythmia, retrograde Purkinje potentials (rPP) were carefully mapped during sinus rhythm in patients with documented VT. Intracardiac signals from a representative case are illustrated in Fig. 2.

In 21/27 (78%) patients, only a retrograde approach was used, in 3/27 (11%) patients, a retrograde and an additional transeptal approach, and in another 3/27 (11%) patients, solely a transeptal approach. Mean procedure time was 130 ± 49 min. Nine of the 27 (33%) patients underwent ablation in sinus rhythm due to non-inducibility. Mean number of RF applications was 12 ± 13 (Table 2), and in all patients, focal ablation sets were performed.

**Table 2:** Procedural characteristics

Patients	*N* = 27
LPF origin, *n* (%)	21 (77.8%)
PVCs/ns-VTs	9/21 (42.9%)
VTs	12/21 (57.1%)
LAF origin, *n *(%)	4 (14.8%)
PVCs/ns-VTs	3/4 (75%)
VTs	1/4 (25%)
US origin, *n* (%)	2 (7.4%)
PVCs/ns-VTs	1/2 (50%)
VTs	1/2 (50%)
Inducibility (Isoprenaline and PES), *n* (%)	16 (59.3%)
Isoprenaline	13 (48.1%)
PES	3 (11.1%)
QRS Duration, mean ± SD, msec	123.8 ± 15.2
Retrograde access only, *n* (%)	21 (77.8%)
Retrograde + transeptal access, *n* (%)	3 (11.1%)
Transeptal access only, *n* (%)	3 (11.1%)
Mean procedure time ± SD, min	130 ± 48.9
Radiofrequency applications, mean ± SD	11.8 ± 12.6

*LPF* left posterior fascicle, *LAF *left anterior fascicle, *UP *upper septal, *PVCs *premature ventricular complexes, *ns-VTs *non-sustained ventricular tachycardia, *VTs *ventricular tachycardia, *PES *programmed electrical stimulation, *SD *standard deviation

### Acute results and repeat ablations

Ablation was acutely successful in 24 patients (89%). In two patients (7%), no ablation was performed due to inability to induce VT under sedation in spite of application of isoprenaline. Three of the 24 patients (12%) underwent re-ablation due to arrhythmia recurrence shortly after the index procedure. Moreover, the additional three patients underwent repeated ablations due to a different arrhythmia during follow-up. Two patients (7.4%) presenting with structural heart disease remained on anti-arrhythmic medications post ablation. No procedure-related minor or major complication occurred. After ablation, 12-lead ECG showed a left fascicular posterior block in 4/24 patients (16.7%) and a complete left bundle block in 1 patient (4.2%), which did not lead to LV dysfunction over long-term follow-up.

### Long-term outcomes

**Fig. 3:**
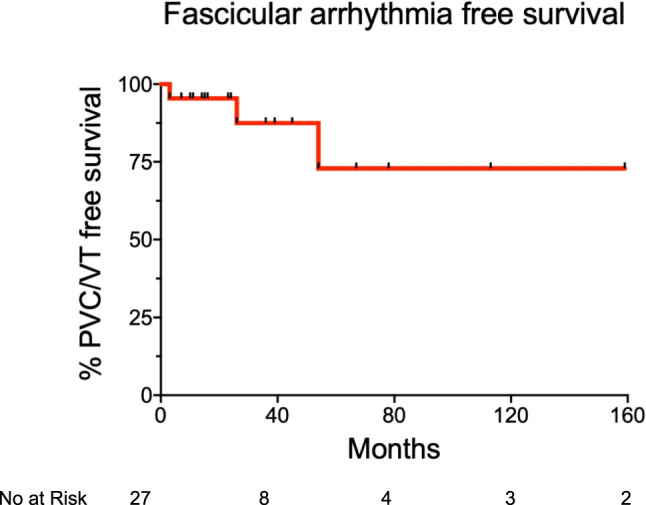
Long term follow-up. Kaplan–Meier survival analysis after catheter ablation of fascicular ventricular arrhythmias. PVC, premature ventricular complex; VT, ventricular arrhythmia

Among the 27 patients included in this study, 8 (30%) were lost to follow-up. The overall median follow-up duration was 30 (IQR 13.5–61.5) months. Kaplan–Meier estimated a freedom from PVCs/(ns)-VTs of 73% at 13-year FU (Fig. 3). Patients who experienced recurrence had significantly longer procedure durations compared to those without recurrence (159 ± 47 vs. 113 ± 43 min, p = 0.03), likely reflecting increased difficulty in substrate mapping. Additionally, we observed a trend toward differing substrate localization between the two groups. In the recurrence group, LPF localization was less frequent, with a shift toward LAF and US localizations (62.5% LPF, 12.5% LAF, 12.5% US), compared to the success group (84.2% LPF, 10.5% LAF, 5.2% US). P1 and P2 timing did not differ significantly between groups. However, clearly identifiable early retrograde Purkinje potentials during mapping in sinus rhythm were more frequently observed in the success group (39% vs. 25%). Despite these trends, no clinical or procedural parameter emerged as a definitive statistical predictor of recurrence.

## Discussion

### Main findings

In this study we found that (i) the anatomical localization of ventricular arrhythmias along the left ventricular conduction system is associated with varying degrees of severity. Arrhythmias along the posterior fascicle more commonly presented as sustained VTs, while those along the anterior fascicle were more frequently associated with PVCs/ns-VTs. Upper septal VT localization was rare, and in our cohort, it had an equal incidence of PVCs/ns-VTs and VTs; (ii) ablation performed at a tertiary center is both safe and effective; and (iii) it is associated with a favorable long-term outcomes (Central Illustration).

### Clinical implications

This study examined both acute and long-term outcomes of patients undergoing catheter ablation for not only VTs, but also PVCs and ns-VTs involving the left ventricular conduction system.

The arrhythmia entity—whether as PVCs, ns-VTs, or sustained VTs—differed according to the anatomical localization along the LV conduction system, as outlined above. The acute success rate in our cohort after the index procedure was 89%, with a cumulative long-term success rate of 72.9%. This is slightly lower than what has been reported in the literature for fascicular VTs, where success rates often exceed 90% [13–15]. The discrepancy could be attributed to the inclusion of a broader spectrum of arrhythmias involving the conduction system in our study, not limited to VTs. No procedural related complications occurred, in particular no deaths or need of urgent cardiac surgery were reported, in line with previous literature data [13]. Ablation was associated with a mild risk of fascicular injury which had no clinical significance or adverse sequelae over long-term follow-up.

Overall, our study presents one of the largest case series on this topic reported in Europe, offering valuable insights into the efficacy and safety of ablation procedures across this diverse patient cohort.

### Limitations

This study has several limitations. It is a retrospective, single-center study. Like most of the studies published, evidence in this field is limited to small non-randomized observational studies. This is likely due to the low prevalence of such arrhythmias, especially in western countries. Additionally, multiple operators were involved in the procedures, employing slightly different ablation approaches. Finally, multielectrode catheters (e.g., HD-Grid, PentaRay) were not systematically used, which could have provided higher-fidelity mapping and better visualization of Purkinje signals.

## Conclusion

Ablation of arrhythmias originating in proximity to the left ventricular conduction system in a tertiary ablation center is safe, effective, and results in a high single procedural success rate over a long-term follow-up.

## Data Availability

The data supporting the findings of this study are available from the corresponding author upon reasonable request.
